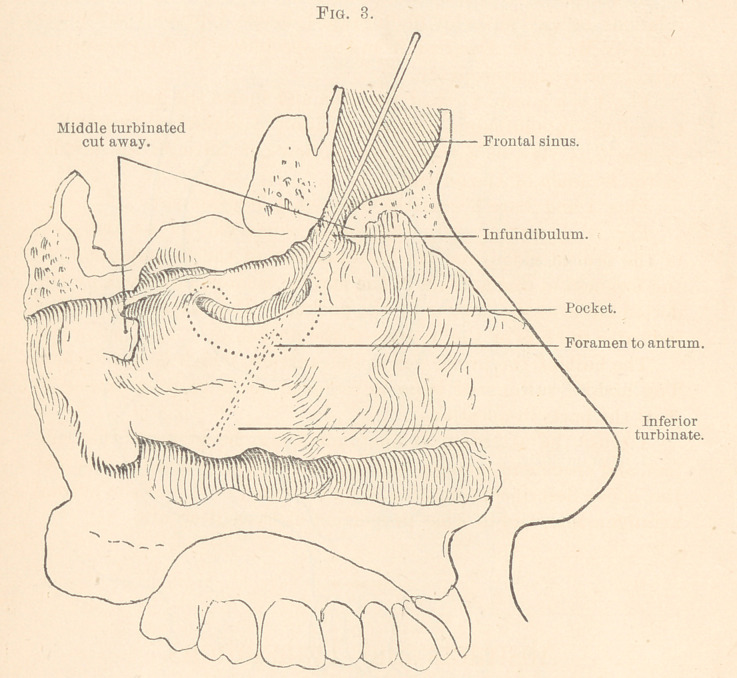# A Study of the Relation of the Frontal Sinus to the Antrum

**Published:** 1897-01

**Authors:** Thomas Fillebrown

**Affiliations:** Boston, Mass.


### A STUDY OF THE RELATION OF THE FRONTAL SINUS TO THE ANTRUM.[1]

BY DR. THOMAS FILLEBROWN, BOSTON, MASS.

My attention was especially called to the relation of the frontal sinus some four years ago, by difficulties I met in inducing a cure in several different cases of empyema of the antrum. In each of these cases the frontal sinus was plainly involved and seemed to be connected with the cause of the antral trouble.

Several of my troublesome cases were sent me by a specialist in the treatment of the nose and throat, whose skill they had defied for over two years.

The paper described five cases, in all of which the conditions of the frontal region showed plainly there was inflammation in the frontal sinus.

One patient had suffered from a disagreeable discharge from the nose for nine years; one for three years, and others for two years or more.

In each case pus continued to gather in the antrum, even when, after washing out thoroughly, examination showed an apparently healthy condition of the lining membrane of the cavity.

Each of these cases I treated by making a good-sized opening through the region of the tooth-sockets, thus affording drainage from the dependent parts of the cavities. In each case teeth had been lost entirely, or only roots remained, so no teeth had to be sacrificed to accommodate the operations.

---

[1] Abstract from a paper read before the American Dental Association, Saratoga, August 5, 1895.

Antrum number one was entered through the socket of the second bicuspid.

Number two, through the second molar region.

In number three the absence of the first bicuspid afforded opportunity.

Number four was entered through the sockets of the first molar, and number five through the location of the second molar.

I made the openings as large as a common lead-pencil, and made a hard rubber plug attached by a clasp to a neighboring tooth to keep the artificial canal patulous, and render the atmospheric condition normal. The plugs could easily be removed by the patient, and after cleaning be as readily replaced.

I have tried both the open tube and the solid plug, and much prefer the latter. It is hardly practical to use a canula large enough to syringe through freely and not to also allow the circulation of air, which is not a natural condition.

The plug being easily removed, both plug and cavity can be washed thoroughly clean. A tube cannot be made clean without much trouble.

The results in these cases are as follows:

Number three cured. Has been entirely well over a year. Number five has steadily gained and is nearly well. Number four improved for a year, then sickened and died from other troubles. Number two, of nine years' standing, keeps himself comfortable, with steady but slow improvement. Number one keeps himself comfortable by daily syringing of the cavity. In each case with a probe wound with cotton I could explore the whole cavity and locate any pus-producing spots, or any collection of secretions; and in cases numbers one and five, by passing the probe through the foramen into the nose, I constantly found pus, which was secreted at that point or came down from the sinus above, which I have since found to be entirely probable, as I shall show later.

I have never found any difficulty in inducing a cure of empyema of the antrum in a few weeks when the cause was of purely dental origin. This being the fact, and the frontal sinus in these cases being so evidently affected, led me to conclude that there must be a very much more intimate relation between the two cavities than that described by anatomist or surgeon, for I could find neither an anatomist or surgeon who could give me the least encouragement that my surmise was correct.

During the past winter I succeeded in verifying my opinion. Professor Dwight, of the Harvard Medical Faculty, kindly offered me an opportunity to examine several specimens in the Harvard Anatomical Museum, and enabled me to obtain others especially for my purpose, and gave me access to his extensive library.

I believed the infundibulum had some direct connection with the antrum, and discharged its secretions directly into it, and an examination of eight different specimens showed that to be the case.

The infundibulum, instead of terminating in the middle meatus, continues as a half-tube, and this half-tube terminates directly in the foramen of the maxillary sinus. This was the case in all of the eight specimens, and in seven of the specimens there is a fold of

Fig. 1.

mucous membrane which serves as a continuation of the unciform process and reaches upward, covering the foramen and forming a pocket which effectually prevents any secretion from the frontal sinus getting into the meatus until the antrum and pocket are full to overflowing.

The pocket I have mentioned has been noticed by a few writers, but has been considered by them as an anomaly. If an anomaly, it is remarkable that I should have found it in seven out of eight specimens obtained at random, and the eighth specimen, in which it is absent, is plainly abnormal, as the foramen is very large and very irregular.

FIG. 2.

The continuation of the infundibulum is present in every specimen, and if the pocket is abnormal, my examinations show that it exists often enough to presume it present in every case where the frontal sinus is affected in conjunction with the antrum, and the discharge from the antrum will not cease.

As I remarked before, few have mentioned the physiological connection of the cavities.

Professor Dwight says, in answer to my request, "I have looked the matter up, and am convinced that the infundibulum opens most directly into the antrum, and that the common opening of the two into the middle meatus is practically on the inner side of the infundibulum."

Tillaux points out "that if fluid be injected into the frontal sinus, instead of running into the middle meatus, it passes in great

Fig. 3.

part into the antrum," and Merkle describes a fold of mucous membrane under the common opening, and accounts by this for the occurrence described by Tillaux.

Dr. W. H. Cryer mentions in his valuable paper read before this Association last year, that fluid may enter the antrum from the frontal sinus, but he makes no mention of the intimate connection which I have observed.

Professor Harrison Allen, in a paper published in the *Dental*

*Cosmos*, 1895, discusses the proliferation of empyema of the frontal sinus into the antrum, and of their coexistence in these cavities.

Dr. J. H. Bryan, in a paper published in the *Transactions of the American Laryngological Association*, 1895, mentions the fact of occasional communication between the two sinuses, but considers them anomalies.

Further than this I find no mention of this condition.

As the parts so overlay each other, it is impossible to show their relations by any series of photographs, hence I present drawings made by Mr. J. W. Emerton from the specimens in my possession, which verify their accuracy.

Fig. 1 is a lateral view of the face, and shows the general direction of the infundibulum, and the position of the antral foramen.

Fig. 2 is view of a transverse section cut through the middle of the foramen of the antrum.

Figs. 1 and 2 are necessarily somewhat diagrammatic.

Fig. 3 shows correctly the frontal sinus cut off at the level of the orbital ridge, and the continuation of the infundibulum to the foramen of the antrum in the middle meatus, indicated by the dotted circle at the bottom of the pocket. The curved dotted line indicates the line of the bottom of the pocket.

The bulb of the probe lies in the antrum, which is not opened. The middle turbinated bone is removed so as to fully expose to view the parts in question.

I trust the attention of anatomists may be given to this subject, and specimens enough may be examined to determine whether the above-described condition is an anomaly, or one of the normal arrangements, and in what proportion of cases it occurs.

## Figures and Tables

**Fig. 1. f1:**
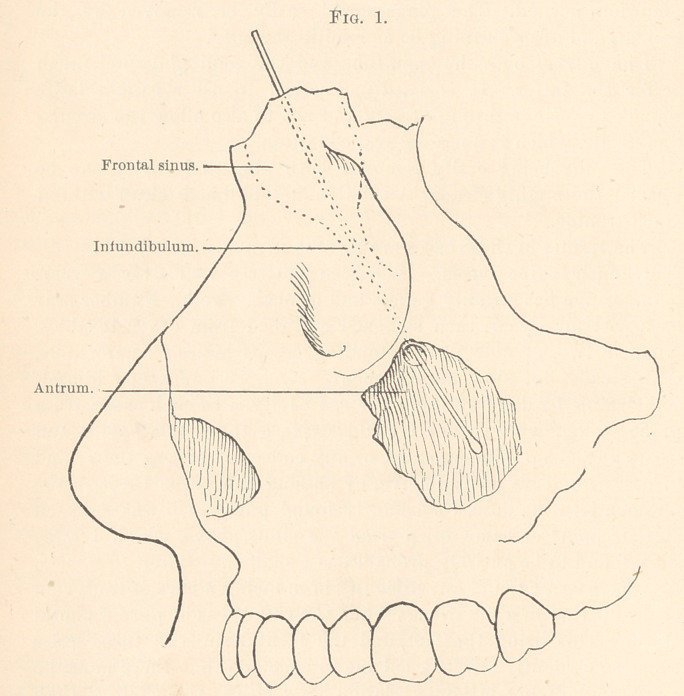


**Fig. 2. f2:**
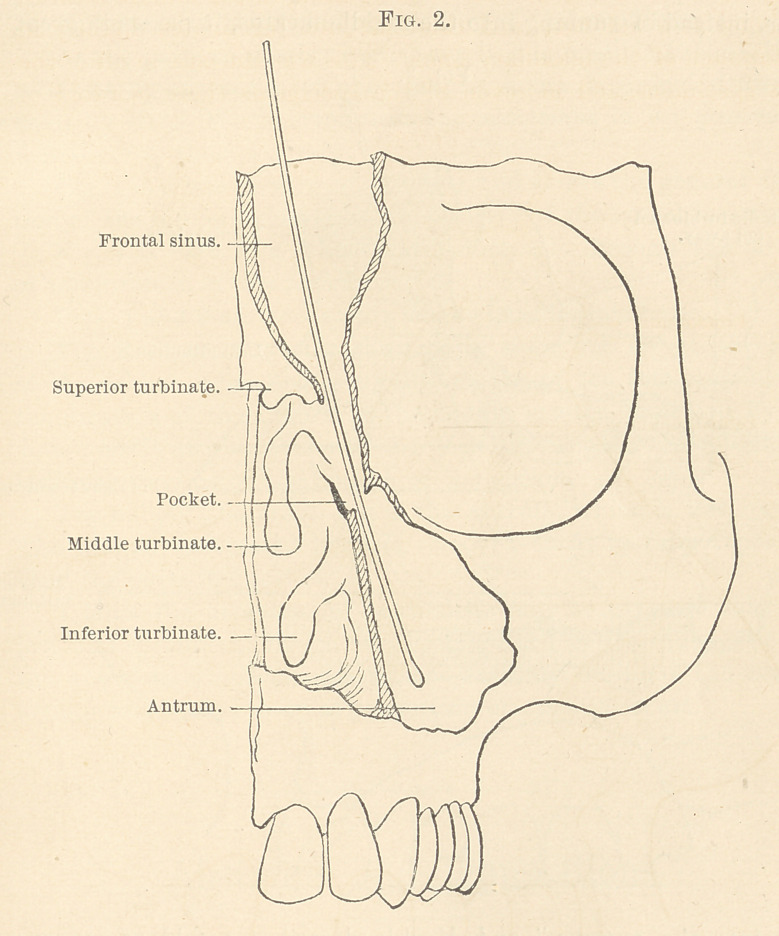


**Fig. 3. f3:**